# Patient-Facing Mobile Apps to Treat High-Need, High-Cost Populations: A Scoping Review

**DOI:** 10.2196/mhealth.6445

**Published:** 2016-12-19

**Authors:** Karandeep Singh, Kaitlin Drouin, Lisa P Newmark, Malina Filkins, Elizabeth Silvers, Paul A Bain, Donna M Zulman, Jae-Ho Lee, Ronen Rozenblum, Erika Pabo, Adam Landman, Elissa V Klinger, David W Bates

**Affiliations:** ^1^ Departments of Learning Health Sciences and Internal Medicine University of Michigan Medical School Ann Arbor, MI United States; ^2^ Department of Pediatric Newborn Medicine Brigham and Women’s Hospital Boston, MA United States; ^3^ Information Systems Partners HealthCare System Wellesley, MA United States; ^4^ University of Massachusetts Medical School Worcester, MA United States; ^5^ Harvard Medical School Boston, MA United States; ^6^ Division of General Medical Disciplines Stanford University School of Medicine Stanford, CA United States; ^7^ Center for Innovation to Implementation VA Palo Alto Health Care System Menlo Park, CA United States; ^8^ Department of Emergency Medicine, Asan Medical Center University of Ulsan College of Medicine Seoul Republic of Korea; ^9^ Division of General Internal Medicine Brigham and Women’s Hospital Boston, MA United States; ^10^ Department of Emergency Medicine Brigham and Women’s Hospital Boston, MA United States; ^11^ Department of Health Policy and Management Harvard TH Chan School of Public Health Boston, MA United States

**Keywords:** review, mobile apps, mHealth, chronic disease, self-management

## Abstract

**Background:**

Self-management is essential to caring for high-need, high-cost (HNHC) populations. Advances in mobile phone technology coupled with increased availability and adoption of health-focused mobile apps have made self-management more achievable, but the extent and quality of the literature supporting their use is not well defined.

**Objective:**

The purpose of this review was to assess the breadth, quality, bias, and types of outcomes measured in the literature supporting the use of apps targeting HNHC populations.

**Methods:**

Data sources included articles in PubMed and MEDLINE (National Center for Biotechnology Information), EMBASE (Elsevier), the Cochrane Central Register of Controlled Trials (EBSCO), Web of Science (Thomson Reuters), and the NTIS (National Technical Information Service) Bibliographic Database (EBSCO) published since 2008. We selected studies involving use of patient-facing iOS or Android mobile health apps. Extraction was performed by 1 reviewer; 40 randomly selected articles were evaluated by 2 reviewers to assess agreement.

**Results:**

Our final analysis included 175 studies. The populations most commonly targeted by apps included patients with obesity, physical handicaps, diabetes, older age, and dementia. Only 30.3% (53/175) of the apps studied in the reviewed literature were identifiable and available to the public through app stores. Many of the studies were cross-sectional analyses (42.9%, 75/175), small (median number of participants=31, interquartile range 11.0-207.2, maximum 11,690), or performed by an app’s developers (61.1%, 107/175). Of the 175 studies, only 36 (20.6%, 36/175) studies evaluated a clinical outcome.

**Conclusions:**

Most apps described in the literature could not be located on the iOS or Android app stores, and existing research does not robustly evaluate the potential of mobile apps. Whereas apps may be useful in patients with chronic conditions, data do not support this yet. Although we had 2-3 reviewers to screen and assess abstract eligibility, only 1 reviewer abstracted the data. This is one limitation of our study. With respect to the 40 articles (22.9%, 40/175) that were assigned to 2 reviewers (of which 3 articles were excluded), inter-rater agreement was significant on the majority of items (17 of 30) but fair-to-moderate on others.

## Introduction

Caring for high-need, high-cost (HNHC) populations represents a complex problem because these individuals often suffer from multiple chronic conditions, functional limitations, behavioral health problems, socioeconomic challenges, and inadequate coordination of care [1,2]. Nearly half of all US adults suffer from a chronic illness and this group accounts for a large share of health care costs [3]. Advances in mobile phone technology coupled with increased availability and adoption of mobile health apps have changed the landscape of self-management [4]. Data increasingly support the role of patient-facing health information technology tools in improving patient-centered care outcomes, health services efficiency, and health outcomes [5-7]. Community health centers and clinics that care for vulnerable populations overwhelmingly perceive mobile health technologies as an ideal tool to engage their patient populations in chronic disease management [8].

Although more than 165,000 mobile health apps are available on the iTunes (iOS) and Google Play (Android) app stores in the Unites States [9] and billions of dollars are being invested in digital health [10], it is not clear how many of these apps focus on patients with chronic conditions and how well the scientific evidence supports their effectiveness. Prior reviews of the literature evaluating the use of patient-facing health apps have been limited by a narrow scope. Reviews have focused on a single medical condition [11-13], on a single aspect of a broad group of apps (such as identifying target populations, behavioral functionalities, privacy policies, and expert involvement) [14-17], or have included only clinical trial–based evidence [9,18], which represents a minority of the ongoing research. A recent systematic review of apps targeting diabetes mellitus, cardiovascular disease, and lung disease found only 3 studies in which a chronic disease management app was used as an intervention and a clinical outcome was measured [19]. Another review focused on how apps can be leveraged by nonprofessional caregivers to care for patients [20].

Although clinical trial evidence supporting the use of apps is generally lacking, this finding may be explained by several factors. First, health apps are fairly new as a medium for engaging patients in comparison with other digital media; therefore, research supporting their use may be ongoing but not yet published. If that is the case, evidence may be found in “gray literature” such as conference proceedings that has not yet made its way to peer-reviewed journals. Second, app developers may be participating in and using research findings to market their apps, which may favor obtaining lower-quality evidence because it is less costly and potentially biased toward a favorable result. Third, it is possible that high-quality evidence exists but that prior reviews failed to uncover it because they focused too narrowly on a small set of disease areas. Given this set of limitations, a “scoping review” may better describe the extent and quality of the literature as well as evidence gaps in comparison with a traditional systematic review [21].

To address the need for a comprehensive assessment of health app evidence, we performed a scoping review in order to (1) assess the breadth of app coverage across HNHC populations, (2) characterize the quality of the published literature (including full-length journal articles and work presented at scientific conferences), (3) evaluate the possibility of biases due to conflicts of interest, and (4) evaluate the types of outcomes measured.

## Methods

### Data Sources and Searches

Studies that evaluated health-related apps for mobile devices were identified by searching PubMed and MEDLINE (National Center for Biotechnology Information), EMBASE (Elsevier), the Cochrane Central Register of Controlled Trials (EBSCO), Web of Science (including the Conference Proceedings Citation Indexes; Thomson Reuters), and the NTIS (National Technical Information Service) Bibliographic Database (EBSCO). The search was conducted between June 20, 2014, and July 14, 2014. The complete search strategy including search terms is available in Multimedia Appendix 1. Our search was designed to identify studies examining applications or software programs running on mobile devices such as mobile phones or tablets that are designed to address the health-related needs of specific HNHC populations. Populations included in the search were older adults (age ≥65 years); individuals with chronic conditions including coronary artery disease, congestive heart failure, hypertension, stroke, chronic obstructive pulmonary disease, cancer, diabetes mellitus, obesity, arthritis, chronic kidney disease, cirrhosis, organ transplantation, or chronic pain; the psychologically or mentally vulnerable who have been diagnosed with depression, bipolar disease, posttraumatic stress disorder, attention-deficit hyperactivity disorder, autism, substance-related disorders, dementia, cognitive impairment, developmental delays, or mental impairment; individuals with medication management issues (multiple medications); individuals with physical handicaps or disabilities, including the blind and deaf; and the socially vulnerable including those with low literacy or numeracy, limited English proficiency, minority status (Native American, Hispanic, African American), low income or homelessness, or infection with human immunodeficiency virus.

**Figure 1:**
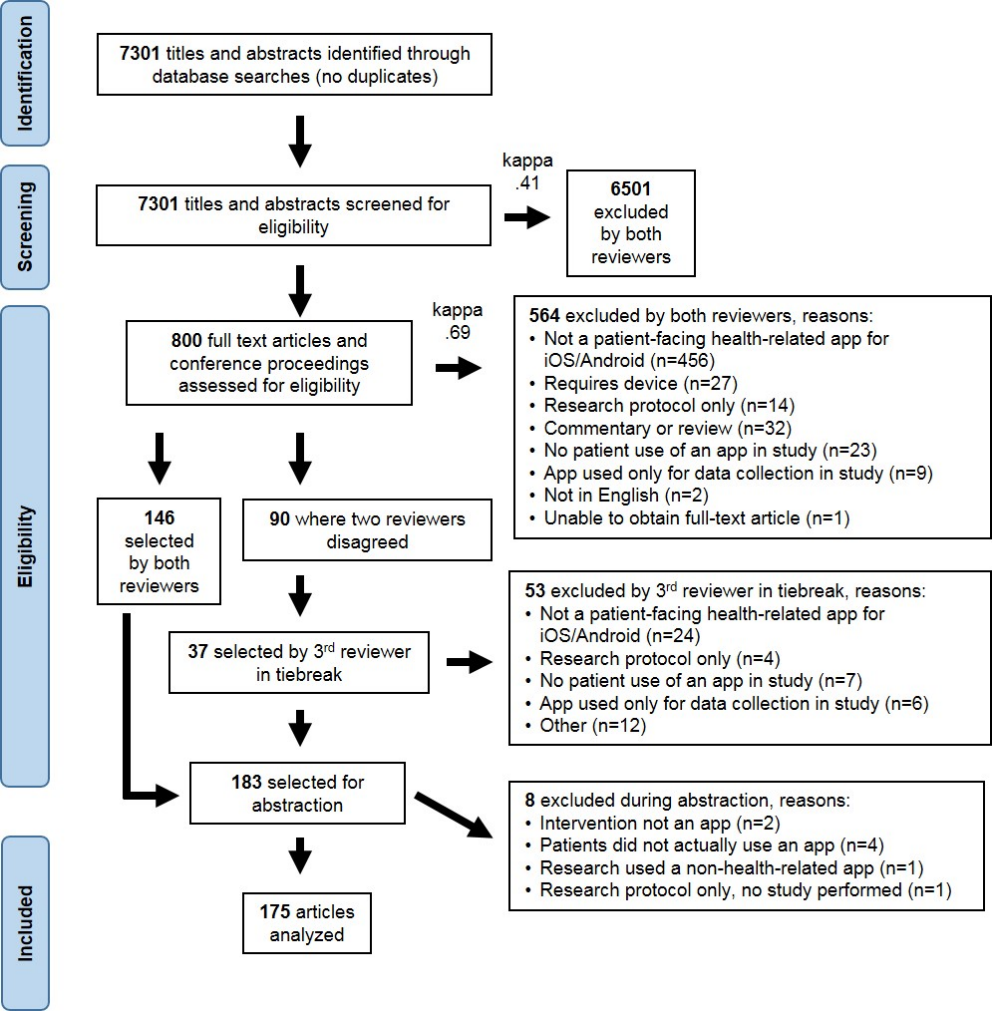
Article selection process.

Appropriate controlled vocabulary terms were included when available (Medical Subject Headings and Emtree). The retrieval set was limited to articles published in 2008 or later; this start date was selected to coincide with when the iOS and the Android app stores were established. No language restrictions were applied, although non-English abstracts were excluded during title and abstract review (Figure 1). Articles not pertaining to native iOS and Android apps were excluded during the full manuscript review.

### Study Selection

All titles and abstracts were individually examined by 2 reviewers (KS and KD, or KS and LPN). Abstracts were included if they described original research written in the English language involving use of an iOS- or Android-based health-related patient-facing mobile app by study subjects. Patient-facing apps are those intended for use primarily by patients or their caregivers. We selected articles that described either iOS or Android apps because the 2 operating systems serve different demographics, with lower-income individuals, blacks, and younger adults preferring Android devices [22]. Articles describing apps focused on supportive technologies (eg, hearing or vision aids), communication technologies (eg, apps used to help autistic children communicate in school settings), or apps requiring a medical device (eg, an app to interact with artificial pancreas) were excluded. Study design was not a basis for exclusion. Full-length articles were obtained for all abstracts identified for inclusion by *either* reviewer. Certain included abstracts could not be linked to full-length manuscripts because they were associated with conference proceedings, including oral presentations or poster sessions; these abstracts were included despite the absence of a full-length manuscript as we wanted to capture gray literature in our review.

The full-length manuscripts and conference abstracts were evaluated by 2 reviewers (MF and ES) to confirm that they met the inclusion criteria. Articles identified for inclusion by *both* reviewers were selected for abstraction. Articles where the 2 reviewers disagreed were evaluated by a third reviewer (KD) to break ties.

### Data Extraction and Quality Assessment

An abstraction survey tool was created to capture information about both the mobile app as described in the publication and the study itself, including the characteristics of the studied apps, quality of evidence, presence of conflict of interest, and types of outcomes evaluated. During a pilot phase, 8 study investigators each abstracted 3 articles using the tool (24 articles in total); changes were made based on feedback until there was consensus regarding the face validity of the tool.

Abstraction of the selected articles was then performed by 1 reviewer (MF or ES). A total of 40 randomly selected articles were evaluated by both reviewers to assess the level of agreement (Table 1).

Details regarding the abstracted items are presented in Multimedia Appendix 2. App engagement was assessed using a previously described framework [23]. We evaluated the following areas for each article:

#### General

We captured information about the app studied, including its target population, platform, availability on the app store, and functionalities to support patient engagement.

#### Quality of the Evidence

We ascertained factors that influence quality and generalizability of the article, including study design, enrollment, follow-up, role of the app in the context of the intervention, and inclusion of relevant patient populations.

#### Declaration of Conflicts

We determined whether any members of the research team were developers of the app in question (or in a formal role supporting app development such as the advisory board) or whether the app developer directly funded the research. While a conflict of interest does not invalidate the results of a given study, literature written or funded by a company responsible for the product being researched is known to be systematically biased [24].

#### Outcomes Evaluated

We evaluated the outcomes considered by each study and assessed their direction (ie, positive, neutral, or negative). Clinical outcomes were those directly related to patient care (eg, decreased hemoglobin A_1c_). Safety or adverse event outcomes were those relevant to unintended negative consequences of an app. Usability outcomes were those describing an app’s ease of use—in some usability studies, multiple rounds of testing are performed, in which case the direction of the outcome was classified based on the final round of testing. Usage describes the amount of time users engaged with the app—this was not reported in a standard fashion and therefore we based the direction on the authors’ expectations, considering “sufficient usage” if observed usage matched expectations. Process outcomes refer to measures pertaining to actions taken in response to the app (eg, undergoing testing for hemoglobin A_1c_)—because the result of the action is not considered (eg, decreased hemoglobin A_1c_), this is not a clinical outcome. A validation outcome was considered present when an app focused on measurement (eg, an app for assessing hepatic encephalopathy) was compared with a non–app-based clinical measure. We evaluated whether the app-based measure performed differently from a non–app-based clinical measure (eg, asterixis). If the article also used a gold standard test (eg, neuropsychiatric testing), we ascertained whether the app-based measure was better or worse than the non–app-based clinical measure. The user satisfaction outcome referred simply to whether users were satisfied with an app.

### Data Synthesis and Analysis

Data from the reviewers were imported into R version 3.2.2 (R Foundation for Statistical Computing). Descriptive statistics were calculated and accompanied by a narrative summary.

## Results

### Article Selection and Abstraction

We identified 7301 titles and abstracts, of which 800 were identified for inclusion by either reviewer (Figure 1). Two reviewers evaluated the full-length manuscripts and 146 articles were selected by both reviewers for abstraction. Of the 90 articles identified for inclusion by only 1 reviewer, a third reviewer selected 37 for abstraction, resulting in a total of 183 articles being selected. During the abstraction process, 8 articles were identified as not meeting the inclusion criteria. After examination by a second reviewer, consensus was achieved on all 8 articles to exclude from the analysis. Thus, in total, 175 articles were abstracted (Multimedia Appendix 3).

Of the 40 articles (22.9%, 40/175) randomly selected for evaluation by both reviewers, 3 were excluded after further examination. As a result, 37 articles were abstracted by both reviewers, and the level of agreement was generally good, with some exceptions such as patient engagement and whether caregivers were included as subjects (Table 1).

**Table 1:** Level of agreement on items on the abstraction form.

Question^a^		Kappa
**General**		
	Who is the primary population(s) that would benefit from the app studied?	.79^b^
	Which platform(s) is used by the app(s) mentioned in the study?	1.0^b^
	Is the app(s) studied currently available on the iTunes or Google Play app store?	.85^b^
	Based on the app's description in the article, how does it engage patients?	.26^b^
	Did the app link to a medical device in the study (eg, glucometer)?	1.0
	Did the app link to a consumer wearable device in the study?	1.0
**Quality of evidence**		
	What is the study design?	.62^b^
	How many total subjects are enrolled in this study (including controls for controlled trials)?	.72
	Is the app studied a standalone intervention (or are there multiple interventions concurrent with app use)?	.59
	What was the average length of follow-up reported (in months)?	.54
	Was this associated with a conference proceeding (abstract, poster, presentation, etc)?	.93
	Does the study have a clinicaltrials.gov registration number?	1.0
	Was at least one of the above vulnerable populations included as subjects in the study?	.49
	Does the study include children as subjects (people under 18 years old)?	.85
	Does the study include people aged 65 or older as subjects?	.79
	Were caregivers for at least one of the above vulnerable populations included as subjects in the study?	.29
**Conflict of interest**		
	Did the research team or their employer contribute to the design or development of the app?	.52
	What is the source of external funding for this study?	.35^b^
**Outcomes evaluated**		
	Was a clinical outcome considered in this study?	.43
	If yes, in what direction was the clinical outcome with use of the app?	.36
	Was a safety or adverse event outcome (caused by the use of the app) considered in the study?	0^c^
	Was a usability outcome considered in the study?	.53
	Was a usage outcome considered in the study?	.71
	If yes, in what direction was the usage outcome with use of the app?	.61
	Was a process measure considered in this study?	.41
	If yes, in what direction was the process measure with use of the app?	.39
	Was a validation outcome considered in this study?	.80
	If yes, in what direction was the validation outcome with use of the app?	.69
	Was user satisfaction considered in this study?	.84
	If yes, in what direction was the satisfaction outcome with use of the app?	.71

^a^See Multimedia Appendix 2 for additional information regarding the questions.

^b^Items where reviewers could select multiple options. Only perfect agreement was considered agreement in the kappa calculation.

^c^There was only 1 article evaluated by 2 reviewers in which 1 reviewer marked safety or adverse event outcome as being present.

### Identification of Overlapping Research

Of the 175 selected articles, we found 15 sets of articles that assessed the same app, in some instances using different study designs, numbers of participants, or end points. For the purposes of our analysis, we considered each article as separate. We found 2 articles that evaluated the ActiveLifestyle app [25,26], AsthmaCare [27,28], EncephalApp [29,30], iMigraine [31,32], iStepLog [33,34], the Mayo Clinic app [35,36], Multiple Sclerosis Performance Test [37,38], My Meal Mate [39,40], Ready~Steady [41,42], a cognitive stimulation app for alcoholics [43,44], USMART [45,46], ClinTouch [47,48], and a food addiction intervention [49,50]. We found 3 articles that evaluated a mobile application in the Women with Epilepsy: Pregnancy Outcomes and Deliveries (WEPOD) study [51-53] and 3 that evaluated the SaGAS 20/10 app [54,55].

### Characteristics of the Mobile Apps

Of the 27 vulnerable populations targeted by the literature search, the groups most commonly targeted by apps included individuals with obesity, physical handicaps, diabetes, older age, and dementia or mild cognitive impairment (Table 2).

**Table 2:** Primary population that would benefit from the app studied.

Population^a^	Number of articles (N=175), n (%)
Obesity	24 (13.7)
Physical handicap or disability (including blindness or deafness)	19 (10.9)
Diabetes mellitus	15 (8.6)
Older adults	15 (8.6)
Dementia or mild cognitive impairment	14 (8.0)
Cancer	11 (6.3)
Autism spectrum disorder	10 (5.7)
Alcohol or drug abuse	7 (4.0)
Chronic pain	7 (4.0)
Depression	7 (4.0)
Coronary artery disease	6 (3.4)
Schizophrenia or psychosis	5 (2.9)
Arthritis	4 (2.3)
Stroke	4 (2.3)
Cirrhosis	3 (1.7)
Congestive heart failure	3 (1.7)
Hypertension	3 (1.7)
Posttraumatic stress disorder	3 (1.7)
Developmentally delayed or mentally impaired	2 (1.1)
HIV^b^ or AIDS	2 (1.1)
Attention-deficit hyperactivity disorder	1 (0.6)
Bipolar disorder	1 (0.6)
Chronic kidney disease	1 (0.6)
Low income or poor	1 (0.6)
Low literacy or low numeracy	1 (0.6)
Posttransplant	1 (0.6)
Smoking	1 (0.6)
None of the above	38 (21.7)

^a^These are not mutually exclusive categories. Articles may evaluate multiple apps and individual apps may target multiple populations.

^b^HIV: human immunodeficiency virus.

Of the 175 selected articles, 60.6% (106/175) involved iOS apps, 32.0% (56/175) involved Android apps, and 7.4% (13/175) involved both. Reviewers evaluated the availability of these apps on both the iTunes (iOS) and Google Play (Android) app stores. Reviewers were unable to search for the app being studied in 40.0% (70/175) of the articles because the name of the app was not mentioned. Apps from an additional 29.7% (52/175) articles were searched and unable to be found on either app store. Among the articles where an app was found, 66% (35/53) were found on the iOS app store, 6% (3/53) on the Android app store, and 28% (15/53) on both. The ways in which apps engaged patients were assessed based on the functionality described in the articles (Table 3).

**Table 3:** How health apps engage patients.

Type of engagement^a^	Number of articles (N=175) n (%)
Records information	132 (75.4)
Provides guidance	64 (36.6)
Displays a patient’s health information	55 (31.4)
Reminds or alerts patients	45 (25.7)
Provides educational information	36 (20.6)
Enables data sharing with clinician	36 (20.6)
Enables data sharing with caregiver	15 (8.6)
Engages through social media	14 (8.0)
Not enough information to determine	10 (5.7)
None of the above	6 (3.4)

^a^These are not mutually exclusive categories. Apps may engage patients in multiple ways.

The most common functionalities were recording information, providing guidance, and displaying health information, and the least common were engaging with social media and enabling communication with family members. A total of 6 (3.4%, 6/175) articles described apps with the ability to connect with a medical device and 5 (2.9%, 5/175) described apps able to connect with a consumer wearable device.

### Quality of Evidence

The method of dissemination involved full-text publications for 136 articles and conference proceedings (eg, oral or poster presentation) for 39 articles. Cross-sectional studies accounted for 42.9% (75/175) of studies (Table 4). Methodologies with lower bias were represented as follows: randomized controlled trials (10.3%, 18/175), nonrandomized controlled trials (2.9%, 5/175), and prospective cohort studies (21.7%, 38/175). The median number of participants in the studies was 31 (interquartile range, IQR, 11.0-207.2, maximum 11,690). The median length of follow-up for non–cross-sectional studies—weighted for the number of participants when articles involved multiple substudies—was 1.4 months (IQR 0.6-3, maximum 42.6).

**Table 4:** Study designs used in abstracted articles.

Study design^a^	Number of articles (N=175) n (%)
Cross-sectional study	75 (42.9)
Prospective cohort study	38 (21.7)
Qualitative research	34 (19.4)
Before-after study	22 (12.6)
Randomized controlled trial	18 (10.3)
Nonrandomized controlled trial	5 (2.9)
Case report or case series	3 (1.7)
Randomized trial with no control	1 (0.6)
Interrupted time series	1 (0.6)
Not enough information to determine	1 (0.6)

^a^These are not mutually exclusive categories. Articles may use multiple study designs or may describe multiple substudies.

Among 26 studies with a control arm, the app was the sole intervention in 21 (81%, 21/26) articles and just one part of a multipart intervention in 5 (19%, 5/26) articles. In the remaining 149 articles, all study subjects were exposed to the app. Only 7.4% (13/175) studies were registered on ClinicalTrials.gov. Of the 137 articles for which a specific population was identified that may benefit from the app (eg, patients with heart disease), 78.1% (107/137) included members of that particular population in the study; 13.1% (18/137) of studies focused on screening or prevention and therefore participants were healthy individuals; the remaining 8.8% (12/137) of studies did not include participants from the relevant population.

Of the 175 studies, 38 (21.7%, 38/175) studies included children, 53 (30.3%, 53/175) included adults aged 65 years or older, and 17 (9.7%, 17/175) included caregivers of HNHC patients.

### Declaration of Conflicts

The authors of the 175 identified studies or their employer directly contributed to the design or development of the app in 107 (61.1%, 107/175) articles; among these, however, 5 (2.9%, 5/175) did not state this explicitly in the body of the paper. The authors of the identified studies were not involved in app development in 28 (16.0%, 28/175) articles, and in 40 (22.9%, 40/175) we were unable to confirm the presence or absence of involvement. Of the 175 studies, 61 (34.9%, 61/175) studies were funded by a government agency, 41 (23.4%, 41/175) by a nonprofit organization, 12 (6.9%, 12/175) by a for-profit company, and 6 (3.4%, 6/175) by a medical professional society; 5 (2.9%, 5/175) studies reported they had no external funding. No statement about the funding source was present in 82 (46.9%, 82/175) articles.

### Types of Outcomes Evaluated

Among the 175 articles, 87 (49.7%, 87/175) articles evaluated user satisfaction, finding users to be generally satisfied in 74 (85%, 74/87), generally unsatisfied in 2 (2%, 2/87), and neutral in the remaining 11 (13%, 11/87) articles. A total of 74 (42.3%, 74/175) articles evaluated usability—often multiple cycles of testing were described, and the first cycle typically had worse performance than later cycles after modifications were made. A total of 61 (34.9%, 61/175) articles looked at usage, finding “sufficient” usage in 53 (87%, 53/61) and lower-than-expected use in 8 (13%, 8/61). Of the 175 articles, 56 (32.0%, 56/175) articles validated the measurement ability of apps in comparison with a clinical measure, finding the app to perform better than the clinical measure in 6 (11%, 6/56), worse than the clinical measure in 17 (30%, 17/56), and no different from the clinical measure in 34 (61%, 34/56) studies. A total of 40 (22.9%, 40/175) studies assessed a process measure (eg, increased administration of smoking cessation counseling), as opposed to a clinical outcome (eg, decreased rate of lung cancer). Of these, there were 35 (88%, 35/40) studies with improvement, 1 (2%, 1/40) with worsening, and 4 (10%, 4/40) with no change in the process outcome. A total of 36 (20.6%, 36/175) articles evaluated clinical outcomes, with 26 (72%, 26/36) demonstrating improvement in clinical outcomes and 10 (28%, 10/36) with no change. Only 9 (5.1%, 9/175) articles considered a safety or adverse event outcome caused by use of the app.

## Discussion

### Principal Findings

While there is optimism that mobile health apps may support the health of HNHC populations, existing research does not robustly evaluate this potential. Our review of the evidence supporting patient-facing mobile health apps identified a number of gaps in the current body of research. A few HNHC groups (older adults and people with obesity, physical handicaps, diabetes, and dementia) are more commonly studied, and we found less than 10 studies published for 20 of the 27 HNHC groups included in our review. The majority of apps studied were unavailable to consumers, the study designs were primarily cross-sectional, non–cross-sectional studies had a fairly short length of follow-up, and study sizes were small. In most cases, developers were often the ones evaluating the apps, sample sizes were small, funding sources were ambiguous, and clinical outcomes were evaluated in a minority of studies. Even among high-quality studies, drawing an inference about the usefulness of an app was frequently limited by intervention arms in which the app was a small piece of a much larger intervention.

Some of the methodological problems we identified such as small sample sizes and short length of follow-up could be addressed if apps incorporated the consent process and data collection into the apps’ functionality. Many of the studies used a traditional “in-person” consent process in order to enroll study subjects. While this may conform to the standards of traditional clinical research, using this approach may limit the number of subjects who can be enrolled and the length of follow-up. New methodological approaches that enable large-scale app outcomes research are needed [56]. Controlled trials where the consent process and data collection occur entirely in the context of a publicly available app may enable such work. The barrier to entry for integrating research into apps has been lowered by frameworks such as Apple ResearchKit, which was used to enroll 11,000 participants for a cardiovascular study in 24 hours [57].

### Recommendations

On the basis of our findings, we make the following recommendations for researchers undertaking the study of mobile apps for the purposes of dissemination:

First, the researchers should consider evaluating apps in understudied HNHC groups to address the current imbalance in the body of research between HNHC groups. Second, reports should include the name of the app or intervention, so that literature about the app can be linked to it definitively; every effort should be made to include a bundle ID, permanent app store weblink, or other unique identifier to facilitate identification of the app. Third, researchers conducting interventional studies should consider the inclusion of both a control arm and an app-only intervention arm to make clearer the link between the app and the outcome. Fourth, studies should clearly state the nature of the relationship between the study contributors and the app developers; if the researchers are also the app developers, researchers should consider validating their work at an additional site supervised by a nondeveloper. Fifth, studies should clearly state the funding source or note if no external funding was used. Finally, researchers should report negative results.

In addition, funders will need to support additional evaluations of apps and should target evaluations that target clinically important outcomes and are large enough to deliver meaningful results. With newer enrollment approaches, it may be possible to enable much larger clinical trials, which may be feasible at low expense because much of the data usually collected may be extracted from existing electronic health records.

We used a robust multistage scoping review process involving 2 reviewers in most steps. We included gray literature in our analysis through a search of conference proceedings and did not limit our analysis to only high-quality evidence.

### Limitations

Although 2 reviewers participated in the process of screening abstracts and full-length manuscripts for eligibility, data abstraction was carried out by only 1 reviewer for most studies. While the majority (17 of 30) of abstracted items had good agreement among the reviewers, interrater agreement was moderate (kappa .41-.6) for 7 items and fair (kappa .21-.40) for 6 questions, which limits the reliability of conclusions drawn from them. To put the kappa values into context, 2 reviewers agreed on ascertainment of a clinical outcome (kappa .43) on 31 out of 37 articles. In particular, the questions with the lowest interrater agreement involved determining whether safety or adverse event outcomes were considered, whether caregivers were included the study, how the app mentioned in the study engaged patients, and the funding source. The low agreement levels for these questions may be attributed to the heterogeneity in the detail to which studies reported information about the apps and study conducted, which is partly due to the inclusion of conference abstracts in our analysis. The agreement was moderate when reviewers abstracted the types of outcomes measured in the studies. We attribute this partly to the breadth of populations that we considered because what constitutes a clinical outcome differs significantly between chronic conditions. Additionally, differentiating clinical outcomes from process outcomes carries some subjectivity and may introduce disagreements. We did revise our abstraction form based on a review of 24 articles during a pilot phase, but additional cycles of revision may have further improved interrater agreement.

We did not evaluate articles that were not in English, which limits our generalizability toward apps targeting non-English speakers. Finally, we conducted our literature search in 2014, which does not capture recent trends.

### Recent Trends in the Literature

Recent studies of patient-facing apps have provided supporting evidence for the role of apps in several areas. In a 2016 randomized controlled crossover study of a mobile app focused on supporting drug intake and vital sign documentation, researchers found that patients who used the iPad app showed greater adherence for both medication intake and blood pressure measurement than a paper-based control group [58]. Another randomized controlled study published in 2016 found that overweight and obese adults who used a social support app lost on average 3 kg more than patients using a self-monitoring control app over the course of the study [59]. Evidence from other recent trials has demonstrated the ability of apps to reduce consumption of sugar-sweetened beverages in women and nutrient-poor foods in men, increase activity level and reduce fatigue following stroke, and improve respiratory parameters with a reduction in corticosteroid usage among individuals with uncontrolled asthma [60-62].

### Conclusions

In the future, providers may routinely prescribe apps for their HNHC patients, and health care systems may invest in them. However, given the limited availability of high-quality evidence for most of the HNHC groups included in our review, we would not expect systematic reviews or meta-analyses focused on these groups individually to yield enough evidence to assess the effectiveness of disease-specific apps. Additionally, apps are being lost in translation from research to the app stores, resulting in a lack of commercial impact of existing research. Despite these limitations, the body of evidence overwhelmingly reports early results that favor the use of mobile health apps.

## Appendix

Multimedia Appendix 1 Search Terms.

Multimedia Appendix 2 Abstraction Form.

Multimedia Appendix 3 Articles included in review (n=175) and complete results of abstraction.

## Abbreviations

HNHC: high-need, high-cost
IQR: interquartile range
