# Supplementary material for: Patient-Facing Mobile Apps to Treat High-Need, High-Cost Populations: A Scoping Review
Source: JMIR Mhealth Uhealth. 2016 Dec 19;4(4):e136. doi: 10.2196/mhealth.6445 (PMC5206484; doi:10.2196/mhealth.6445)
Supplement: Multimedia Appendix 1 [file mhealth_v4i4e136_app1.pdf]

Supplemental Material

Appendix Exhibit 1: Search Terms

Note: Items in brackets pertain to PubMed.

The groups below were searched as:
#1 AND (#2 OR #3 OR #4 OR #5)

Mobile Apps (#1)

"Mobile Applications"[Mesh] OR "Cellular Phone"[mesh] OR "Computers, Handheld"[mesh] OR ((app[tiab] OR apps[tiab] OR application*[tiab] OR technology[tiab] OR platform*[tiab] OR computer program*[tiab] OR software[tiab]) AND (smartphone*[tiab] OR phone[tiab] OR phones[tiab] OR tablet*[tiab] OR handheld*[tiab] OR iphone*[tiab] OR ipad*[tiab] OR android*[tiab])) OR mobile app*[tiab] OR mobile technolog*[tiab] OR mobile device*[tiab] OR mobile comput*[tiab]

Elderly (#2)

"Aged"[Mesh] OR late life[tiab] OR elder*[tiab] OR aged patient*[tiab] OR old patient*[tiab] OR older patient*[tiab] OR older person*[tiab] OR old people[tiab] OR older people[tiab] OR oldest people[tiab] OR older adult*[tiab] OR oldest adult*[tiab] OR geriatric*[tiab] OR aging[tiab] OR age 65[tiab] OR age 70[tiab] OR age 75[tiab] OR age 80[tiab] OR senior*[tiab]

Chronic Conditions (#3)

"Coronary Artery Disease"[Mesh] OR coronary artery disease*[tiab] OR coronary atherosclero*[tiab] OR coronary arteriosclero*[tiab] OR "Heart Failure"[Mesh] OR heart failure[tiab] OR "Pulmonary Disease, Chronic Obstructive"[Mesh] OR chronic obstructive pulmonary disease[tiab] OR copd[tiab] OR chronic bronchitis[tiab] OR emphysema[tiab] OR "Hypertension"[Mesh] OR hypertensi*[tiab] OR high blood pressure[tiab] OR elevated blood pressure[tiab] OR "Stroke"[Mesh] OR stroke*[tiab] OR cerebrovascular accident*[tiab] OR "Neoplasms"[Mesh] OR neoplasm*[tiab] OR cancer*[tiab] OR carcinoma*[tiab] OR sarcoma*[tiab] OR adenocarcinoma*[tiab] OR leukemia*[tiab] OR lymphoma*[tiab] OR melanoma*[tiab] OR "Diabetes Mellitus"[Mesh] OR diabet*[tiab] OR "Obesity"[mesh] OR obesity[tiab] OR obese[tiab] OR "Arthritis"[Mesh] OR arthriti*[tiab] OR osteoarthriti*[tiab] OR "Renal Insufficiency, Chronic"[Mesh] OR chronic kidney[tiab] OR chronic renal[tiab] OR "Liver Cirrhosis"[Mesh] OR cirrhosis[tiab] OR "Chronic Pain"[Mesh] OR pain[tiab] OR "Transplantation"[Mesh] OR transplant*[tiab] OR transplantation*[tiab]

Mental Health Disorders and Physical Disabilities (#4)

"Depressive Disorder"[Mesh] OR "Depression"[Mesh] OR depression[tiab] OR depressive[tiab] OR "Bipolar Disorder"[Mesh] OR bipolar[tiab] OR manic[tiab] OR "Schizophrenia and Disorders with Psychotic Features"[mesh] OR psychosi*[tiab] OR psychose[tiab] OR psychoses[tiab] OR psychoti*[tiab] OR schizophren*[tiab] OR schizoaffect*[tiab] OR schizotyp*[tiab] OR "Stress Disorders, Traumatic"[Mesh] OR stress disorder*[tiab] OR ptsd[tiab] OR posttraumatic stress*[tiab] OR traumatic stress*[tiab] OR stress symptom*[tiab] OR "Attention Deficit Disorder with Hyperactivity"[Mesh] OR attention deficit*[tiab] OR adhd[tiab] OR "Autistic Disorder"[Mesh] OR autistic*[tiab] OR autism*[tiab] OR "Substance-Related Disorders"[Mesh] OR "Smoking"[Mesh] OR "Smoking Cessation"[Mesh] OR drug abuse*[tiab] OR drug addict*[tiab] OR drug dependen*[tiab] OR substance dependen*[tiab] OR substance abuse*[tiab] OR illicit drug[tiab] OR drug use*[tiab] OR alcohol*[tiab] OR drinking[tiab] OR drinker*[tiab] OR amphetamine*[tiab] OR methamphetamine*[tiab] OR methylenedioxyamphetamine[tiab] OR nmda[tiab] OR ecstasy[tiab] OR cocaine[tiab] OR marijuana[tiab] OR cannabis[tiab] OR opiate*[tiab] OR opioid*[tiab] OR heroin[tiab] OR morphine[tiab] OR smoking[tiab] OR smoker*[tiab] OR tobacco[tiab] OR cigar*[tiab] OR "Dementia"[Mesh] OR dementia*[tiab] OR alzheimer*[tiab] OR lewy bod*[tiab] OR "Mild Cognitive Impairment"[Mesh] OR cognitive impairment*[tiab] OR impaired cogniti*[tiab] OR cognitive decline*[tiab] OR memory impair*[tiab] OR impaired memor*[tiab] OR "Intellectual Disability"[mesh] OR mentally impaired[tiab] OR mental impairment*[tiab] OR mentally retarded[tiab] OR mental retardation[tiab] OR mental deficienc*[tiab] OR mentally def*[tiab] OR intellectual disabilit*[tiab] OR intellectually disabled[tiab] OR mentally disabled[tiab] OR mental disabilit*[tiab] OR mental def* OR mentally deficient[tiab] OR "Developmental Disabilities"[Mesh] OR developmentally delayed[tiab] OR developmental delay*[tiab] OR developmental disabilit*[tiab] OR developmentally disabled[tiab] OR developmental disorder*[tiab] OR "Reminder Systems"[mesh] OR "Patient Compliance"[mesh] OR "Medication Reconciliation"[mesh] OR ((medication*[tiab] OR patient*[tiab]) AND (adherence[tiab] OR nonadherence[tiab] OR non-adherence[tiab] OR compliance[tiab] OR noncompliance[tiab] OR non compliance[tiab])) OR medication management[tiab] OR medicine management[tiab] OR medication reconciliation[tiab] OR medicine reconciliation[tiab] OR medication reminder*[tiab] OR multiple medication*[tiab] OR multiple medicine*[tiab] OR multiple prescription*[tiab] OR reminder*[tiab] OR "Disabled Persons"[Mesh] OR "Paralysis"[Mesh] OR disabled[tiab] OR handicap*[tiab] OR disabilit*[tiab] OR amputee*[tiab] OR paralysis[tiab] OR paralytic*[tiab] OR parapleg*[tiab] OR quadripleg*[tiab] OR hemipleg*[tiab] OR "Blindness"[Mesh] OR "Deafness"[Mesh] OR blind[tiab] OR blindness[tiab] OR sightless[tiab] OR deaf[tiab] OR deafness[tiab] OR hard of hearing[tiab] OR hearing loss*[tiab] OR impaired hearing[tiab] OR hearling impairment*[tiab]

Socioeconomically Disadvantaged (#5)

"Health literacy"[mesh] OR literacy[tiab] OR literate[tiab] OR illiterate*[tiab] OR reading skill*[tiab] OR reading abilit*[tiab] OR numeracy[tiab] OR numerate[tiab] OR innumerate[tiab] OR innumeracy[tiab] OR numerical skill*[tiab] OR "Communication Barriers"[mesh] OR limited english[tiab] OR low english[tiab] OR non-english speak*[tiab] OR poor english[tiab] OR english profic*[tiab] OR language profic*[tiab] OR "Indians, North American"[Mesh] OR native american*[tiab] OR native north american*[tiab] OR american indian*[tiab] OR first nation*[tiab] OR alaska native*[tiab] OR alaskan native*[tiab] OR native alaskan*[tiab] OR "Hispanic Americans"[Mesh] OR hispanic*[tiab] OR latino*[tiab] OR latina*[tiab] OR mexican american*[tiab] OR puerto rican*[tiab] OR spanish american*[tiab] OR cuban american*[tiab] OR "African Americans"[Mesh] OR african american*[tiab] OR blacks[tiab] OR "Poverty"[mesh] OR "Homeless Persons"[mesh] OR low income[tiab] OR indigen*[tiab] OR poverty[tiab] OR poor*[tiab] OR

homeless*[tiab] OR "HIV Infections"[Mesh] OR acquired immunodeficiency syndrome[tiab] OR human immunodeficiency virus[tiab] OR AIDS[tiab] OR HIV[tiab]
