# Supplementary material for: Patient-Facing Mobile Apps to Treat High-Need, High-Cost Populations: A Scoping Review
Source: JMIR Mhealth Uhealth. 2016 Dec 19;4(4):e136. doi: 10.2196/mhealth.6445 (PMC5206484; doi:10.2196/mhealth.6445)
Supplement: Multimedia Appendix 2 [file mhealth_v4i4e136_app2.pdf]

Supplemental Material

Appendix Exhibit 2: Abstraction Form

GENERAL

Who is the primary population(s) that would benefit from the app studied?
Select all that apply (based on the study's context, background, and discussion). For example, if app focuses on cancer patients with depression, select "Cancer" AND "Depression". If none of the populations listed would benefit from the app, select only "None of the Above".

- ☐ Arthritis
- ☐ Attention deficit hyperactivity disorder
- ☐ Autism spectrum disorder
- ☐ Bipolar disorder
- ☐ Cancer
- ☐ Chronic kidney disease
- ☐ Chronic obstructive pulmonary disease (COPD)
- ☐ Chronic pain
- ☐ Cirrhosis
- ☐ Congestive heart failure
- ☐ Coronary artery disease
- ☐ Dementia or mild cognitive impairment

- ☐ Depression
- ☐ Developmentally delayed or mentally impaired
- ☐ Diabetes mellitus
- ☐ Elderly
- ☐ HIV/AIDS
- ☐ Homeless
- ☐ Hypertension
- ☐ Limited English proficiency speaker
- ☐ Low income or poor
- ☐ Low literacy and/or low numeracy
- ☐ Native American, Hispanic, or African American
- ☐ Obesity
- ☐ Physical handicap or disability (including blindness or deafness)
- ☐ Post-transplant
- ☐ Post-traumatic stress disorder
- ☐ Schizophrenia and/or psychosis

- [ ] Alcohol or drug abuse
- [ ] Smoking
- [ ] Stroke
- [ ] None of the above

Which platform(s) is used by the app(s) mentioned in the study?
Answer this only based on the article text -- please do not search the web or app stores.

- [ ] iOS
- [ ] Android

Is the app(s) studied currently available on the iTunes or Google Play app store?
Search both app stores if the app name is provided (Hint: you can Google "[App name] iTunes" or "[App name] Google Play"). If you find an app on the app store with the same name as an app in the study, do your best to determine whether it is referring to the same app and developer. DO THIS EVEN IF THE APP IN THE STUDY WAS FOR A DIFFERENT PLATFORM.

- [ ] iTunes App Store (iOS)
- [ ] Google Play Store (Android)
- [ ] Not currently available on either
- [ ] Could not search -- app name not provided in article

Based on the app's description in the article, how does it engage patients?

- [ ] Provides educational information
- [ ] Instructs patients (app provides guidance or advice based on information you provide it)

- ☐ Records information
- ☐ Displays patient's health information
- ☐ Reminds or alerts patients
- ☐ Enables communication of information with clinician
- ☐ Enables communication of information with family (e.g. caregiver)
- ☐ Through social media
- ☐ None of the above
- ☐ Not enough information to determine

Did the app link to a medical device in the study (e.g. glucometer)?
If an app has the ability to link to a device but this feature is not used in the study, answer "no". Link to look this up: http://www.accessdata.fda.gov/scripts/cdrh/cfdocs/cfPMN/pmn.cfm

- ○ Yes
- ○ No

Did the app link to a consumer wearable device in the study?
If an app has the ability to link to a device but this feature is not used in the study, answer "no".

- ○ Yes
- ○ No

QUALITY OF EVIDENCE

What is the study design?
If the study used more than one design (ONLY if study had multiple parts), check all that apply. You may refer to this resource for descriptions about study design: http://www.ncbi.nlm.nih.gov/books/NBK95280/

- ☐ Cross sectional study (e.g. taking a survey after one-time use of an app)
- ☐ Randomized controlled trial
- ☐ Non-randomized controlled trial
- ☐ Prospective cohort study
- ☐ Retrospective cohort study
- ☐ Before-after study
- ☐ Interrupted time series
- ☐ Qualitative research (e.g. focus group or usability study AFTER participants have used an app)
- ☐ Case-control study
- ☐ Other: ______

How many total subjects are enrolled in this study (including controls for controlled trials)?
If publication contains multiple substudies, sum total participants from RELEVANT substudies. Example: if two substudies were pre-app development and one was an iOS prototype, we would only look at the last substudy.

______

Is the app studied a standalone intervention (or are there multiple interventions concurrent with app use)?
Answer "No" if the app was only one of the parts of a multi-part intervention (e.g. face-to-face visits, phone calls, etc).

- ○ Yes, app is sole intervention (and there is a control arm that did not use app)

- No, multi-part intervention (and there is a control arm that did not use app)
- Cannot tell, study only has one arm or all arms exposed to app

What was the average length of follow-up reported (in months)?
If not reported, enter "-1". If cross sectional study or focus group, enter "0" for no follow-up. If reported in years, multiply by 12 to convert to months. If reported in days, divide by 30 to convert to months. If publication contains multiple substudies, refer to caption of previous question.

Was this associated with a conference proceeding (abstract, poster, presentation, etc)?

- Yes
- No

Does the study have a clinicaltrials.gov registration number?
This should be mentioned in the publication. Do not search clinicaltrials.gov.

- Yes
- No

If yes, what is the clinicaltrials.gov registration number?
Example: NCT00629304

Was at least one of the above vulnerable populations included as subjects in the study?
Look in the methods under "Inclusion/Exclusion criteria" to determine this.

- Yes
- No, purpose of app was screening/prevention

- No, for any other reason
- N/A (Answered "none of the above" to target population)

Does the study include children as subjects (people under 18 years old)?
Look in the methods under "Inclusion/Exclusion criteria" to determine this.

- Yes
- No
- Cannot determine

Does the study include people aged 65 or older as subjects?
Look in the methods under "Inclusion/Exclusion criteria" to determine this.

- Yes
- No
- Cannot determine

Were caregivers for at least one of the above vulnerable populations included as subjects in the study?
Look in the methods under "Inclusion/Exclusion criteria" to determine this.

- Yes
- No
- N/A (Answered "none of the above" to target population)

CONFLICT OF INTEREST

Did the research team or their employer contribute to the design or development of the app?
Examples of this: if a researcher developed the app or if the researcher formally serves on the advisory board for the app developer.

- ◯ Yes, and this is stated in the article
- ◯ Yes, stated on the app store page, app website, or another website using Google search (but not stated in the article)
- ◯ No, developer was not involved
- ◯ Cannot determine

What is the source of external funding for this study?
This information should be contained in the paper itself. Select all that apply.

- ☐ Not stated
- ☐ No external funding
- ☐ For-profit company
- ☐ Medical professional society
- ☐ Other non-profit organization
- ☐ Government agency

OUTCOMES EVALUATED

Was a clinical outcome considered in this study?
E.g. Hemoglobin A1c for diabetes mellitus.

- ◯ Yes
- ◯ No

If yes, in what direction was the clinical outcome with use of the app?
Use your judgment to answer this. If there is no control group or it is a qualitative study, do your best to characterize the overall findings of the authors for this outcome.

- N/A. Answered "No" to previous question.
- 1 (outcome worse with intervention)
- 2 (no change with intervention)
- 3 (better with intervention)

Was a safety or adverse event outcome (caused by the use of the app) considered in the study?
“Caused” means reasonably related to use of the app. Could be either be a safety event that occurred, or it could be something that the authors merely looked for regardless of whether it actually occurred.

- Yes
- No

Was a usability outcome considered in the study?
E.g. Measuring unintended clicks, watching people use an app and figuring out how their intended action differs from the actual action. Can be patient-reported.

- Yes
- No

Was a usage outcome considered in the study?
E.g. Metric for usage of different parts of the app, how many times users logged into the app, how many messages users send through the app

- Yes

○ No

If yes, in what direction was the usage outcome with use of the app?
Use your judgment to answer this. If there is no control group or it is a qualitative study, do your best to characterize the overall findings of the authors for this outcome.

○ N/A. Answered "No" to previous question.

○ 1 (low use – usage was lower than authors' expectations)

○ 2 (sufficient use – usage matched authors' expectations)

Was a process measure considered in this study?
E.g Decreased outpatient visits or phone calls, higher cancer screening rates. These may imply a clinical or cost benefit but do not measure either of these directly.

○ Yes

○ No

If yes, in what direction was the process measure with use of the app?
Use your judgment to answer this. If there is no control group or it is a qualitative study, do your best to characterize the overall findings of the authors for this outcome.

○ N/A. Answered "No" to previous question.

○ 1 (outcome worse with intervention)

○ 2 (no change with intervention)

○ 3 (better with intervention)

Was a validation outcome considered in this study?

E.g. Comparing a depression questionnaire in an app to an existing "gold standard" (such as PHQ-9 questionnaire) in order to validate the app's functionality.

- Yes
- No

If yes, in what direction was the validation outcome with use of the app?
Use your judgment to answer this. If there is no control group or it is a qualitative study, do your best to characterize the overall findings of the authors for this outcome. If publication contains multiple substudies, select for the outcome of latest data.

- N/A. Answered "No" to previous question.
- 1 (worse than non-app-based measure)
- 2 (no different from non-app-based measure)
- 3 (better than non-app-based measure)

Was user satisfaction considered in this study?
E.g Were users happy with the app? Did they feel the app was useful and satisfied their needs?

- Yes
- No

If yes, in what direction was the satisfaction outcome with use of the app?
Use your judgment to answer this. If there is no control group or it is a qualitative study, do your best to characterize the overall findings of the authors for this outcome.

- N/A. Answered "No" to previous question.
- 1 (generally unsatisfied)

- 2 (not satisfied, not unsatisfied)
- 3 (generally satisfied)
